# Recombination and selectional forces in cyanopeptolin NRPS operons from highly similar, but geographically remote *Planktothrix *strains

**DOI:** 10.1186/1471-2180-8-141

**Published:** 2008-08-26

**Authors:** Trine B Rounge, Thomas Rohrlack, Tom Kristensen, Kjetill S Jakobsen

**Affiliations:** 1 University of Oslo, Department of Biology, Centre for Ecological and Evolutionary Synthesis (CEES), 0316 Oslo, Norway; 2 NIVA, Norwegian Institute for Water Research, 0411 Oslo, Norway; 3 University of Oslo, Department of Molecular Biosciences, 0316 Oslo, Norway; 4 University of Oslo, Microbial Evolution Research Group (MERG), 0316 Oslo, Norway

## Abstract

**Background:**

Cyanopeptolins are nonribosomally produced heptapetides showing a highly variable composition. The cyanopeptolin synthetase operon has previously been investigated in three strains from the genera *Microcystis*, *Planktothrix *and *Anabaena*. Cyanopeptolins are displaying protease inhibitor activity, but the biological function(s) is (are) unknown. Cyanopeptolin gene cluster variability and biological functions of the peptide variants are likely to be interconnected.

**Results:**

We have investigated two cyanopeptolin gene clusters from highly similar, but geographically remote strains of the same genus. Sequencing of a nonribosomal peptide synthetase (NRPS) cyanopeptolin gene cluster from the Japanese strain *Planktothrix *NIES 205 (205-*oci*), showed the 30 kb gene cluster to be highly similar to the *oci *gene cluster previously described in *Planktothrix *NIVA CYA 116, isolated in Norway. Both operons contained seven NRPS modules, a sulfotransferase (S) and a glyceric acid loading (GA)-domain. Sequence analyses showed a high degree of conservation, except for the presence of an epimerase domain in NIES 205 and the regions around the epimerase, showing high substitution rates and Ka/Ks values above 1. The two strains produce almost identical cyanopeptolins, cyanopeptolin-1138 and oscillapeptin E respectively, but with slight differences regarding the production of minor cyanopeptolin variants. These variants may be the result of relaxed adenylation (A)-domain specificity in the nonribosomal enzyme complex. Other genetic markers (16S rRNA, *ntc*A and the phycocyanin *cpc*BA spacer) were identical, supporting that these geographically separated *Planktothrix *strains are closely related.

**Conclusion:**

A horizontal gene transfer event resulting in exchange of a whole module-encoding region was observed. Nucleotide statistics indicate that both purifying selection and positive selection forces are operating on the gene cluster. The positive selection forces are acting within and around the epimerase insertion while purifying selection conserves the remaining (major) part of the gene cluster. The presence of an epimerase in the gene cluster is in line with the D-configuration of Htyr, determined experimentally in oscillapeptin E in a previous study.

## Background

Cyanopeptolins are nonribosomally produced peptides with highly variable composition. The general structure of the cyanopeptolin peptide family encompasses 7 amino acids, including the residue 3-amino-6-hydroxy-2-piperidone (Ahp), where the six C-terminal amino acids form a ring [1,2] and the N-terminal amino acid frequently is N-modified. The N-terminal amino acid and all positions in the ring except position 2 (threonine) and position 4 (Ahp) can be occupied by variable amino acids, giving rise to a large number of cyanopeptolin variants [3].

The succession of the modules [4,5] and specificity of A-domain binding pockets in nonribosomal peptide synthetases (NRPSs) [6,7] can give a good prediction of peptide composition and structure. NRPSs do not always perform stringent substrate selection and incorporation [7], thus, relaxed substrate specificity is common in NRPS [6,8,9]. In addition to the common module domains including the adenylation (A)-, condensation (C)- and thiolation (T)-domains, several tailoring domains have been found associated with cyanopeptolin synthetases. Methyltransferases are present in three cyanopeptolin gene clusters from *Anabaena*, *Microcystis *and *Planktothrix *(*apd*, *mcn *and *oci*). Halogenases are found in *apd *and *mcn*, while the tailoring domains responsible for side chain modification of the N-terminal amino acid are unique for each strain (i.e.; formyl transferase in *apd*, sulfotransferase and glyceric acid (GA) transferase in *oci*, absent in *mcn*).

So far, only cyanopeptolin gene clusters derived from the genera *Anabaena *[10], *Microcystis *[11] and *Planktothrix *[12] have been characterized. They share the same basic domain structure but possess unique tailoring genes and A-domain substrate binding pockets, indicating independent evolution of cyanopeptolin genes within each lineage. Sequence identity is high (approximately 80% in the NRPS module coding regions) between *Microcystis *(*mcn*) and *Planktothrix *(*oci*) cyanopeptolin gene clusters. The more thoroughly investigated microcystin gene clusters show higher sequence identity within a genus than between genera. The same is likely to be the case also for the cyanopeptolin genes.

Sequence variation in microcystin synthetase clusters has been investigated within strains of the genera *Microcystis *[13,14] and *Planktothrix *[15]. Modifications and reorganizations due to several recombination events have been reported [14-16], and together with differences in substrate specificity between equivalent A-domains [17-19] are the reason for the different peptide variants.

*Planktothrix *NIVA CYA 116 (NIVA CYA 116), isolated from a Norwegian lake, produces cyanopeptolin-1138 [12] for which the amino acids configurations are unknown. This peptide was found to be highly similar to oscillapeptin E produced by *Planktothrix *NIES 205 (NIES 205), isolated in Japan [20]. Both peptides have the same molecular mass, but slightly different polarities [12]. A different content of L-/D-amino acids in the peptides was suggested as a possible reason for the observed difference [12]. To investigate the genetic basis of the differences between the peptides, we have cloned and sequenced the NIES 205 cyanopeptolin gene cluster and compared it to the previously characterized NIVA CYA 116 gene cluster. This has allowed us to explore NRPS evolution and genetic variations in closely related strains and to investigate to what extent selectional forces operate on these gene clusters.

## Results

### NIVA CYA 116 and NIES 205 have similar but not identical peptide profiles

The major peptides in the two strains consist of HO_3_SO-CH_2_-CH(OMe)-CO-HTyr-Thr-HTyr-Ahp-Ile-Phe(Me)-Ile ([Table 1, Additional file 1 figure 1] and Rounge *et al *[12]). However, spiking experiments (data not shown) revealed a slight difference in polarity between cyanopeptolin-1138 from NIVA CYA 116 [12] and oscillapeptin E from NIES 205 [20]. In contrast to NIVA CYA 116 producing only cyanopeptolins, screening of NIES 205 shows production of additional peptide from other peptide-classes (data not shown).

Several cyanopeptolin variants were also detected in both strains. LC-MS-MS data identified minute amounts of seven cyanopeptolins in NIVA CYA 116, with variation in the first, third, fifth and/or seventh positions compared to cyanopeptolin-1138/oscillapeptin E [Additional file 1 figure 2]. An earlier study has shown that NIES 205 produce oscillapeptin C, D and E, based on spectroscopic analyses including 2D NMR [20]. Our LC-MS-MS analysis of NIES 205 confirmed the production of oscillapeptin D and E, but also identified a cyanopeptolin with the mass 1074, which is found in NIVA CYA 116 as well [Table 1 and Additional file 1 figure 3].

NIVA CYA 116 and NIES 205 produced similar – but not identical – cyanopeptolin variants. The identified NIVA CYA 116 cyanopeptolins were mainly combinations of Hty/Ile/Leu in positions AA1 and AA3 and Ile/Leu/Val in positions AA5 and AA7. Other unidentified apolar amino acid-like residues were detected in position AA3. In contrast, the only variations observed in the NIES 205 peptides were Hty, Ile/Leu and HcAla in position AA3 (Table 1).

**Table 1:** Oci A-domains binding pockets and peptide profiles

	**Binding pockets**	**OciA-A1**	**OciA-A2**	**OciB-A3**	**OciB-A4**	**OciB-A5**	**OciB-A6**	**OciC-A7**
	**NIVA CYA 116**	DLGFTGAVCK	DFWNIGMVHK	DA**QS**MGAIIK	DVENAGVVTK	DAFFLGVTFK	DAWTIAGVCK	DAFFLGVTFK
	**NIES 205**	DLGFTGAVCK	DFWNIGMVHK	DA**EG**MGAIIK	DVENAGVVTK	DAFFLGVTFK	DAWTIAGVCK	DAFFLGVTFK

**NIVA CYA 116**								

**Mass Da**	**Side chain**	**AA 1**	**AA2**	**AA3**	**AA4**	**AA5**	**AA6**	**AA7**

**1138**	HO_3_-SO-CH_2_-CH(OMe)-COH	HTyr	Thr	HTyr	Ahp	Ile	Phe(Me)	Ile
1124	HO_3_-SO-CH_2_-CH(OMe)-COH	HTyr	Thr	HTyr	Ahp	Val	Phe(Me)	Ile
1124	HO_3_-SO-CH_2_-CH(OMe)-COH	HTyr	Thr	HTyr	Ahp	Ile	Phe(Me)	Val
1074	HO_3_-SO-CH_2_-CH(OMe)-COH	HTyr	Thr	Ile/Leu	Ahp	Ile	Phe(Me)	Ile
1010	HO_3_-SO-CH_2_-CH(OMe)-COH	Ile/Leu	Thr	Ile/Leu	Ahp	Ile	Phe(Me)	Ile
1088	HO_3_-SO-CH_2_-CH(OMe)-COH	HTyr	Thr	X	Ahp	Ile	Phe(Me)	Ile
1122	HO_3_-SO-CH_2_-CH(OMe)-COH	HTyr	Thr	Y	Ahp	Ile	Phe(Me)	Ile

**NIES 205**								

**Mass Da**	**Side chain**	**AA 1**	**AA2**	**AA3**	**AA4**	**AA5**	**AA6**	**AA7**

1138*	HO_3_-SO-CH_2_-CH(OMe)-COH	Htyr	Thr	Htyr	Ahp	Ile	Phe(Me)	Ile
1074	HO_3_-SO-CH_2_-CH(OMe)-COH	Htyr	Thr	Ile/Leu	Ahp	Ile	Phe(Me)	Ile
1128**	HO_3_-SO-CH_2_-CH(OMe)-COH	Htyr	Thr	HcAla	Ahp	Ile	Phe(Me)	Ile

The binding pocket residues of the NIVA CYA 116 and NIES 205 A-domains were identified by comparison to the *GrsA-Phe *A-domain (Residue 235, 236, 239, 278, 299, 301, 322, 330, 331, 517). The Oci-A3 binding pockets are different (in grey) between the two strains, and the divergent amino acids are shown in bold. The composition of cyanopeptolins produced by NIVA CYA 116 and NIES 205 and their molecular weights (M+H+) are shown with amino acids correlated with the putative binding pockets. HcAla is 3-(4'-hydroxy-2'-cyclohexenyl) alanine, and X and Y is unidentified amino acid derivates. Mutual peptides in NIVA CYA 116 and NIES 205 are highlighted in dark grey and light grey. See [Additional file 1 figure 1, 2 and 3 for the peptide structure and more details on MS data.* peptide named oscillapeptin E and **peptide named oscillapeptin D

### Comparison of the 205-*oci *and 116-*oci *gene clusters

**Figure 1:**
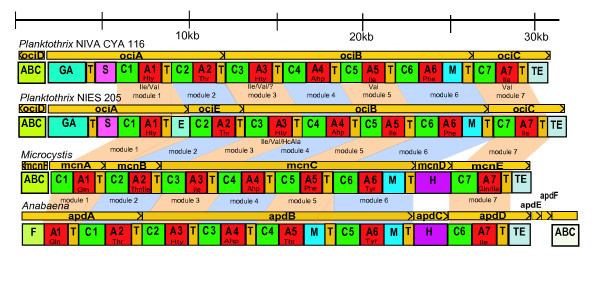
**Comparison of the known cyanopeptolin operons**. The overall structure of cyanopeptolin operons *oci *from *Planktothrix *NIES 205 [GenBank: EU109504] and NIVA CYA 116 [GenBank: DQ837301], *mcn *from *Microcystis *[GenBank: DQ075244] and *apd *from *Anabaena *[GenBank: AJ269505]. Gene names, transcription directions and approximate sizes are indicated above each gene cluster. Adenylation (red), condensation (green), thiolation (yellow), epimerization (turquoise), methyltransferase (blue) sulfotransferase (pink), halogenisation (purple) and termination domains (grey) are shown with their abbreviations. The putative activated amino acids are indicated for each A-domain. Amino acids detected in smaller amounts are beneath the major amino acid. Equivalent modules are depicted in light blue and light orange. The ABC transporter is transcribed in the opposite direction in the *oci *and *mcn *operons, and an ABC transporter is predicted downstream of the *apd *operon.

Anticipating that two strains producing almost identical cyanopeptolins also should contain similar gene clusters, we sequenced a cyanopeptolin gene cluster in NIES 205 (205-*oci*) using primers designed for the cyanopeptolin (*oci*) gene cluster in NIVA CYA 116 (116-*oci*) [12]. The two gene clusters, including the ABC transporter genes and the intergenic spacers, were highly similar (93% identity between the nucleotide sequences), and the domain structures of the encoded synthetases were almost identical; except that 205-*oci *contained an epimerase encoding (E)-domain between T2 and C2 (Figure 1). The position of the E-domain corresponds to the Htyr in D-configuration in oscillapeptin E determined by Itou *et al *[20]. Both gene clusters included a GA-domain and a sulfotransferase domain. Comparison with cyanopeptolin gene clusters characterized in *Microcystis *(*mcn*) [11] and *Anabaena *(*apd*) [10] (Figure 1) showed a higher degree of similarity within the *Planktothrix *genus than between genera (70% identity between OciB and AdpB with the additional methyltransferase excluded and 77% identity between OciC and AdpD). A-domains and A-domain binding pockets signatures were identified from the gene clusters and aligned. The binding pocket signatures in 116-Oci and the corresponding 205-*oci *signatures were identical, except for 116-OciB-A3 (DA**QS**MGAIIK) and 205-OciB-A3 (DA**EG**MGAIIK) (Table 1). Corresponding pairs of 205-Oci and 116-Oci A-domains clustered together in phylogenetic analyses that included A-domains from cyanopeptolin [10-12], microcystin [17,21,22] nostocyclopeptide [23] and nostopeptolide [24] synthetases [Additional file 1 figure 4].

**Figure 2:**
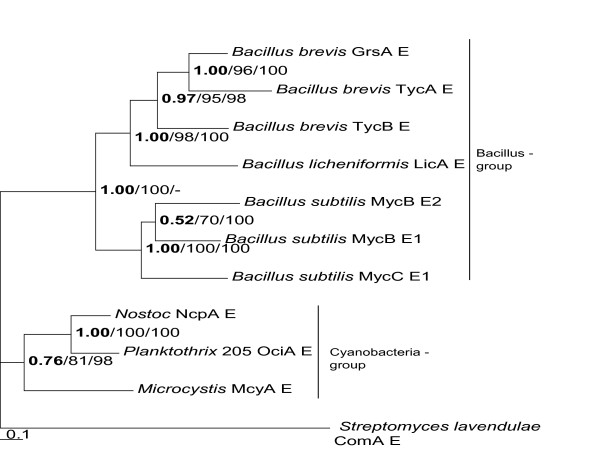
**Phylogenetic analyses of E-domains**. The E-domain phylogenetic tree was constructed utilizing MrBayes 3.1., Wag protein substitution model and gamma-shaped distribution. In addition, the bootstrap obtained for NJ (MEGA 3.1) at default settings and ML (RAxML) trees are indicated. Only posterior probability values and bootstrap replica values above 50% (out of 1000 (NJ) and 100 (ML) trees) are shown.

E-domains are common in cyanobacterial NRPS, found in microcystin, aeruginosin and nostocyclopeptide synthetases, notably, E-domains have until now not been found in cyanopeptolin synthetases. The E-domain produces the D-isomer of the amino acid activated by the upstream A-domain and is also involved in the stereospecific selection of the D-isomer for incorporation in the peptide product. Most E-domains are flanked by T (T_E_)- and C-domains with special motives [25,26], and this was the case also in 205-Oci – as shown by the phylogenetic analyses (see Figure 2).

The NIES 205-E-domain is localized downstream of 205-A1 and T2. A phylogenetic analysis of E-domains (Figure 2), including E-domains from microcystin synthetase (McyA-E) and nostocyclopeptide synthetase (NcpA-E), showed a close relationship between NcpA-E and 205-OciA-E (72% identity on the DNA and 67% similarity on the protein level).

**Figure 3:**
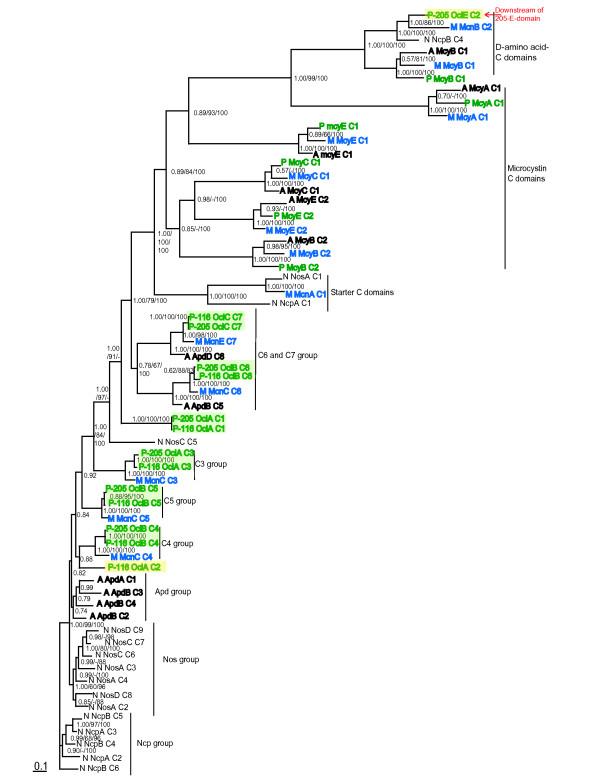
**Phylogenetic analyses of C-domains showing groups according to gene cluster and position/function**. The C-domain phylogeny was constructed using Bayesian inference with gamma distribution, 4 mill generations tree sampling every 100 generations and removal of the first 3000. The topologies generated using NJ (MEGA 3.1) and ML (RAxML) analyses show near identical branching patterns-only minor differences are seen within the Apd group. Bayesian posterior probability, NJ (1000 bootstrap values) and ML (100 trees) above 50% are shown. CpRev protein substitution model was used in the Bayesian and ML analyses. Genus origin is shown with first letter abbreviations (P = *Planktothrix*, M = *Microcystis*, A = *Anabaena *and N = *Nostoc*), and the C-domains are labeled in numerical order according to direction of transcription (i.e. seven *oci*, seven *mcn *and six *apd *C-domains). Corresponding Oci C domains group together, except for C2 situated downstream of the 205-E-domain. C1–C4 *apd*, *nos *and *ncp *C-domains do not group according to function

Phylogenetic analyses of the C-domains (Figure 3), including domains from cyanopeptolin [10-12], microcystin [17,21,22] nostocyclopeptide [23] and nostopeptolide [24] synthetases, clustered according to presence or absence of an upstream E-domain. The 205-Oci-C2-domain grouped with D-amino acid-specific C-domains, while the other 205-Oci-C domains formed a clade with the corresponding 116-Oci-C-domains.

The specialized T_E_-domains associated with E-domains, show major differences within the core T motif compared to standard T-domains [25]. Comparisons of regular T-domains and T_E_-domains, including 205-Oci-T_E_2, McnA-T1 and NcpA-T_E_1-domain, showed an H/D and L/I difference in addition to a gap in the T_E_-domain motif [Additional file 1 figure 5]. N-terminal T-domains, including both 116-T1 and 205-T1, could also be distinguished from T_E_-domains and regular T-domains [Additional file 1 figure 5].

### Other genomic regions confirm a close relationship between the *Planktothrix *strains

Several markers were sequenced to further study the relationship between *Planktothrix *NIVA CYA 116 and NIES 205. The DNA sequences (16S rDNA (1357 bp), a part of *ntcA *(384 bp), a global transcriptional regulator of nitrogen assimilation in cyanobacteria, and the phycocyanin spacer *cpc*BA) displayed 100% identity between the two strains.

### Variation in substitution rates throughout the cyanopeptolin gene clusters

**Figure 4:**
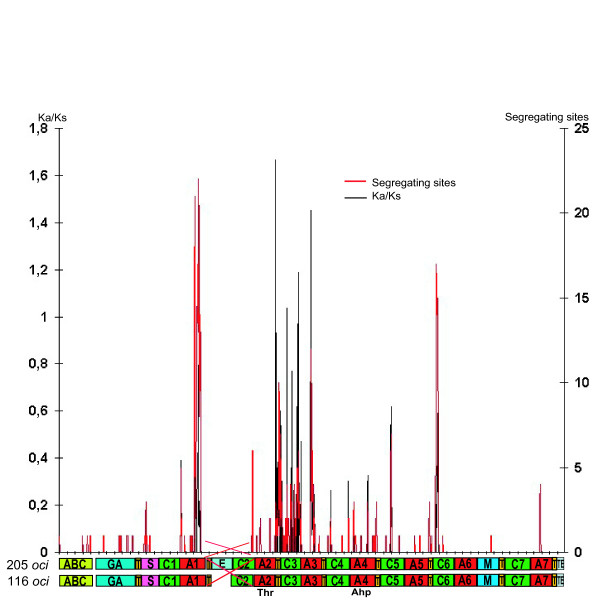
**Distribution of segregating sites and Ka/Ks ratios in the *oci *gene cluster**. The ratios are displayed using the program DnaSP and sliding windows analysis on the alignment of 205-*oci *and 116-*oci*. Window length was 50 bp and step size 10 bp. The distribution of segregation sites (red) and Ka/Ks (black) ratios are shown in correlation with the domain alignment. Module 2 (T2-(E)-C2) has been excluded from the analyses.

Investigation of the substitution rates within the 30 kb 116- and 205-*oci *gene cluster alignment can identify both putative recombination events and regions under specific selection pressure. The region containing the epimerization domain (T2, E, C2) was excluded due to too large overall differences to produce a reliable alignment. Figure 4 shows segregating sites (black lines) and nonsynonymous vs. synonymous substitution rates (red lines) in a sliding window analysis of the alignment. Only a few scattered substitutions can be seen in the first part, containing the ABC transporter, GA, T1, S and C1 domains, and in the last part, containing A6, M, T7, C7, A7, T8 and TE domains. However, the C3 and A3 domains contained several substitutions and the rate of mutations in nonsynonymous sites compared with synonymous sites (Ka/Ks) exceeded 1 – a putative sign of positive selection. A high substitution rate was also observed in a small region in C6 and the last part of A1, but the Ka/Ks ratios did not exceed 1.

## Discussion

### Correlation between cyanopeptolin gene clusters and peptides

The presence of two highly similar NRPS gene clusters (*oci*) in NIVA CYA 116 and NIES 205, and the production of nearly identical peptides by the two strains corroborate the association between the *oci *gene cluster and cyanopeptolin-1138 proposed by Rounge *et al *[12]. This association is further substantiated by high degree of similarity to the cyanopeptolin gene cluster in *Anabaena *(*apd*), where the functional relationship between genes and peptides has been confirmed by a gene knock-out study [10] – as well as similarity to the *Microcystis *cyanopeptolin gene cluster (*mcn*) [11].

### Global dispersal and distribution of cyanopeptolin genes

Based on the genomic regions studied here, two *Planktothrix *strains, NIVA CYA 116 and NIES 205, appear to be closely related despite the geographical separation. This is in accordance with the sequence comparison of 16S rDNA [27] identifying identical 16S rDNA sequences in Japan, China, The Netherlands, UK, Finland, Sweden and Norway, and thus may indicate a global distribution of closely related *Planktothrix *strains. Since Lake Årungen in Norway host international rowing competitions, a co-transport of this *Planktothrix *genotype with rowing equipment may be feasible. The data presented here do not allow any conclusions about global distribution without a more thorough analysis. The highly specific differences observed in the *oci *gene clusters are, independently of geographic distributions, intriguing. Our analyses indicate that the differences to some extent are due to positive selection at specific amino acid positions.

### Variation in peptide content due to lack of specificity in the A-domains?

Previous studies have shown that lack of specificity in A-domains leads to activation of several amino acids with similar properties, thus giving rise to the synthesis of a series of related peptides from a single NRPS system [28]. Ile/Leu/Val activating A-domains have been reported in lichenysin biosynthesis [8], and fengycin synthetase [29] among others. It is likely that the 116-Oci-A5- and A7-domains can activate Leu, Ile and Val and that the 116-Oci-A1- and A3-domains, that mainly activates Htyr, also can activate Ile and Leu. Consequently, 116-Oci is responsible for production of all seven cyanopeptolin detected in NIVA CYA 116 in this study. Likewise, 205-Oci probably is responsible for all oscillapeptin variants. The biological significance of a single NRPS complex giving rise to several peptide variants is yet to be determined.

Six of the seven binding pockets signatures of corresponding A-domains in NIES 205 and NIVA CYA 116 are identical (Table 1). If the different peptide profiles observed in the two strains are due to genetic differences in the NRPS genes, they are likely to be due to differences not involving the amino acids constituting the binding pocket signatures. LC-MS-MS-analyses were performed on strains cultivated on the same media, but we cannot completely exclude substrate availability as a contributory cause of variable peptide amount and peptide profile in the strain.

### Module exchange and amino acid configuration

Over a stretch of total of 30 kb including the ABC transporter, the 116-*oci *and 205-*oci *gene clusters are remarkable similar, except for the modules encoding the T2-(E)-C2 domains. Too low sequence similarity is found between the whole T2-(E)-C2 modules in NIVA CYA 116 and NIES 205 to make a reliable alignment, suggesting that in one of these strains an entire module may have been exchanged through recombination. The E-domain trees (Figure 2) show a close relationship between cyanobacterial E-domains.

Sequence similarity to other E-domains and the distinctive flanking C (Figure 3) and T [Additional file 1 figure 5] domains observed by phylogenetic analysis indicate that the Oci-E-domain is an active epimerase, and are responsible for epimerization of Htyr to D-configuration. The configuration of the amino acids in cyanopeptolin-1138 were not determined however, a D-Htyr in oscillapeptin E and a putative L-Htyr in cyanopeptolin-1138 might explain the small difference between the oligopeptides with regard to polarity observed by HPLC analysis, as reported by Rounge *et al. *[12].

Interestingly, in the corresponding region of the Mcn cyanopeptolin synthetase in *Microcystis *the McnA-T1 and McnB-C2 include motifs suggesting association with an E-domain [11]. In this case, however, no E-domain is present.

### Sequence conservation and selection within cyanopeptolin modules

The two cyanopeptolin gene clusters (205-*oci *and 116-*oci*) are highly similar also at the third codon position. The first part (ABC-transporter, the spacer, GA-, T1-, S-, and C1-domains) and last part (C4-, A4-, T5-, C5-, A5-, T6-, C6-, M-, T7-, C7-, A7-, T8- and TE domains) of the *Planktothrix *cyanopeptolin gene cluster are nearly identical, despite the geographical distance separating the strains. Mechanisms for such sequence conservation may be frequent homology-driven genetic exchange within a genotype, leading to homogenization – in line with the general models suggested by Rudi *et al*. [30], Gogarten *et al*.[31] and Papke *et al*. [32]. Or alternatively sequence conservation may be due to low evolutionary rates caused by purifying selection or very short time of independent evolution.

Analysis of segregating sites and rates of nonsynonymous and synonymous nucleotide substitutions (Ka/Ks) indicate that module 3 (T3-, C3- and A3-domains) is different from the remaining domains by displaying higher substitution rates and signs of positive selection at several sites (Ka/Ks higher than 1). This is the module responsible for incorporation of the amino acid at position AA3 in the peptide.

According to data from Itou *et al *[20], a single amino acid replacement in the AA3 position of oscillapeptin E and F alters the protease inhibitory profile, indicating that this position could be pivotal for the inhibitory activity of cyanopeptolins. Positive selection in the third module could thus be expected to increase the adaptability of the inhibitory- or other putative functions of cyanopeptolin.

## Conclusion

The *Planktothrix *strains of Japan and Norway harbor almost identical cyanopeptolin gene clusters and display very similar (but not identical) cyanopeptolin profiles. The notable gene cluster difference is the presence of an epimerase in NIES 205 corresponding to a D-Htyr in ocillapeptin E. Within a single gene cluster we have demonstrated both positive selection and purifying selection, the first promoting new gene cluster variants following recombination, the latter maintaining a high degree of conservation of the major parts of the gene cluster.

## Methods

### Bacterial cultures

*Planktothrix agardhii *NIVA CYA 116 was isolated in 1983 from Lake Årungen, Norway, and maintained in the NIVA culture collection of Algae. *Planktothrix agardhii *NIES 205 was isolated from Lake Kasumigaura/Ibaraki, Japan in 1982, and maintained in the NIES culture collection [20]. Both strains were cultured in Z8 [33] media at ~20°C with 12 hour illumination at about 15 μmol m^-2 ^s^-1 ^in Sanyo versatile environmental test chamber (FG-4P 36–40).

### PCR and sequencing

DNA from NIES 205 was isolated utilizing Dynabeads (Invitrogen, Carlsbad, USA) [34]. Combinations of PCR primers designed for the cyanopeptolin (*oci*) gene cluster in NIVA CYA 116 [12] were used to amplify regions of a cyanopeptolin gene cluster in NIES 205. These PCR products were sequenced using primer walking. Additional PCR primers were designed to amplify regions between already obtained PCR products. BD Advantage 2 (BD Biosciences, Mountain View, USA) was utilized as polymerase in all PCR amplifications. The PCR products were sequenced using an ABI 3730 sequencer and v3.1 Big Dye solution.

### Sequence analysis and phylogeny

Open reading frames were identified and translated using Vector NTI (Invitrogen, Carlsbad, USA). Domains and their boundaries were identified using the NRPS database [35], A-domain binding pocket residues identified by aligning the sequences with the GrsA-Phe A-domain [6] and substrate specificity predicted utilizing the NRPS database  and phylogenetic analysis. A-, C-, T- and E- domain protein sequences were aligned using MEGA 3.1 and Neighbor-Joining (NJ) trees were constructed using MEGA 3.1 at default settings (Poisson correction as the amino acid substitution model) [36]. Optimal protein evolution model was found by ProtTest [37]. Trees were constructed utilizing MrBayes [38] 3.0 and 3.1 [39] on the UiO Bioportal [40] with an optimal protein substitution model. Variable substitution rates across sites were accounted for by gamma distribution. The MCMC chains were carried out for 4 million generations and trees were sampled every 100 generations, removing 3000 trees before the MCMC chain reached convergence. In addition, maximum likelihood inferences with RAxML [41] were performed on the E- and C- domain alignments. Similarity calculations were done in Vector NTI. DnaSP [42] was used to calculate Ka/Ks ratio and segregating sites with a sliding window with window length of 50 bp and step size 10 bp.

### Mass spectrometry

Freeze-dried material of NIVA-CYA116 and NIES 205 was extracted with 50% MeOH (MeOH:water, v/v) and the extracts were subjected to a screening for cyanopeptolins by LC-MS. The instrument included a Waters Acquity UPLC system equipped with an Atlantis column (C18 2.1 × 150 mm, 5 μm particle size) and set to run a linear gradient starting with 80% solvent A (10 mM ammonium acetate, 0.1% acetic acid) and ending with 60% solvent A after 15 min. Solvent B was MeOH with 0.1% acetic acid. The flow rate was 0.2 ml min^-1^. The LC system was connected to a Waters Quattro Premier XE tandem quadropole mass spectrometer equipped with an electrospray probe. The detector was run in the positive ion mode at a cone voltage of 50 V. A total ion scan from 600 to 1400 Da was performed during the entire length of the LC gradient.

The structures of putative cyanopeptolins were analyzed by MS fragmentation studies. MS fragments hold valuable structural information and have been successfully used before to identify and structurally elucidate cyanobacterial oligopeptides including cyanopeptolins [43-45]. Fragmentation experiments were carried out with the hardware configuration described above. The mass spectrometer was run in daughter ion scanning mode and all settings were automatically optimized for fragmentation at 30 eV. Fragments were recorded during the entire length of the LC gradient. The identification of fragments was assisted by the HighChemMassFrontier software version 3.0. This software predicts MS fragmentation patterns on the basis of a putative structure. Comparing predicted and actual fragmentation patterns was used to assess the accuracy of a putative structure. Further hints to the structure were obtained from the occurrence of typical diagnostic ions such as immonium ions and from predictions on the amino acid occurrence made by the genetic analyses.

## Authors' contributions

This work was performed as part of the PhD thesis for TBR. TBR and TR carried out all experimentation and all authors have contributed to the experimental and analytical design. TBR performed the bioinformatics and phylogenetic analysis under supervision of KSJ and TK. TR carried out the peptide analyses. TBR, KSJ (thesis advisor) TK and TR wrote the ms. All authors have read and approved the final manuscript.

## Supplementary Material

Additional file 1 Peptide structure and NRPS phylogeny. Figure 1: peptide structure of cyanopeptolin 1138. Figure 2: mass spectrometric fragmentation experiments data of NIVA CYA 116. Figure 3: mass spectrometric fragmentation experiments data of NIES 205. Figure 4: NRPS A-domain phylogeny. Figure 5: Sequence analyses of T-domains. Table 1: Accession numbers Click here for file
