# Supplementary material for: Recombination and selectional forces in cyanopeptolin NRPS operons from highly similar, but geographically remote Planktothrix strains
Source: BMC Microbiol. 2008 Aug 26;8:141. doi: 10.1186/1471-2180-8-141 (PMC2533009; doi:10.1186/1471-2180-8-141)
Supplement: Additional file 1 — Peptide structure and NRPS phylogeny. Figure 1: peptide structure of cyanopeptolin 1138. Figure 2: mass spectrometric fragmentation experiments data of NIVA CYA 116. Figure 3: mass spectrometric fragmentation experiments data of NIES 205. Figure 4: NRPS A-domain phylogeny. Figure 5: Sequence analyses of T-domains. Table 1: Accession numbers [file 1471-2180-8-141-S1.doc]

# Additional file 1:

# Peptide structure and NRPS phylogeny

**Figure 1**: Putative peptide structure of cyanopeptolin 1138 produced by *Planktothrix* NIVA CYA 116, almost identical to oscillapeptin E produced by *Planktothrix* NIES 205. Asterisks denote that the chemical analysis cannot distinguish between Ile, Leu, and *allo*-Ile. However, *Planktothrix* NIVA CYA 116 *in silico* analyses clearly suggest Ile at positions 5 and 7 [12].

**
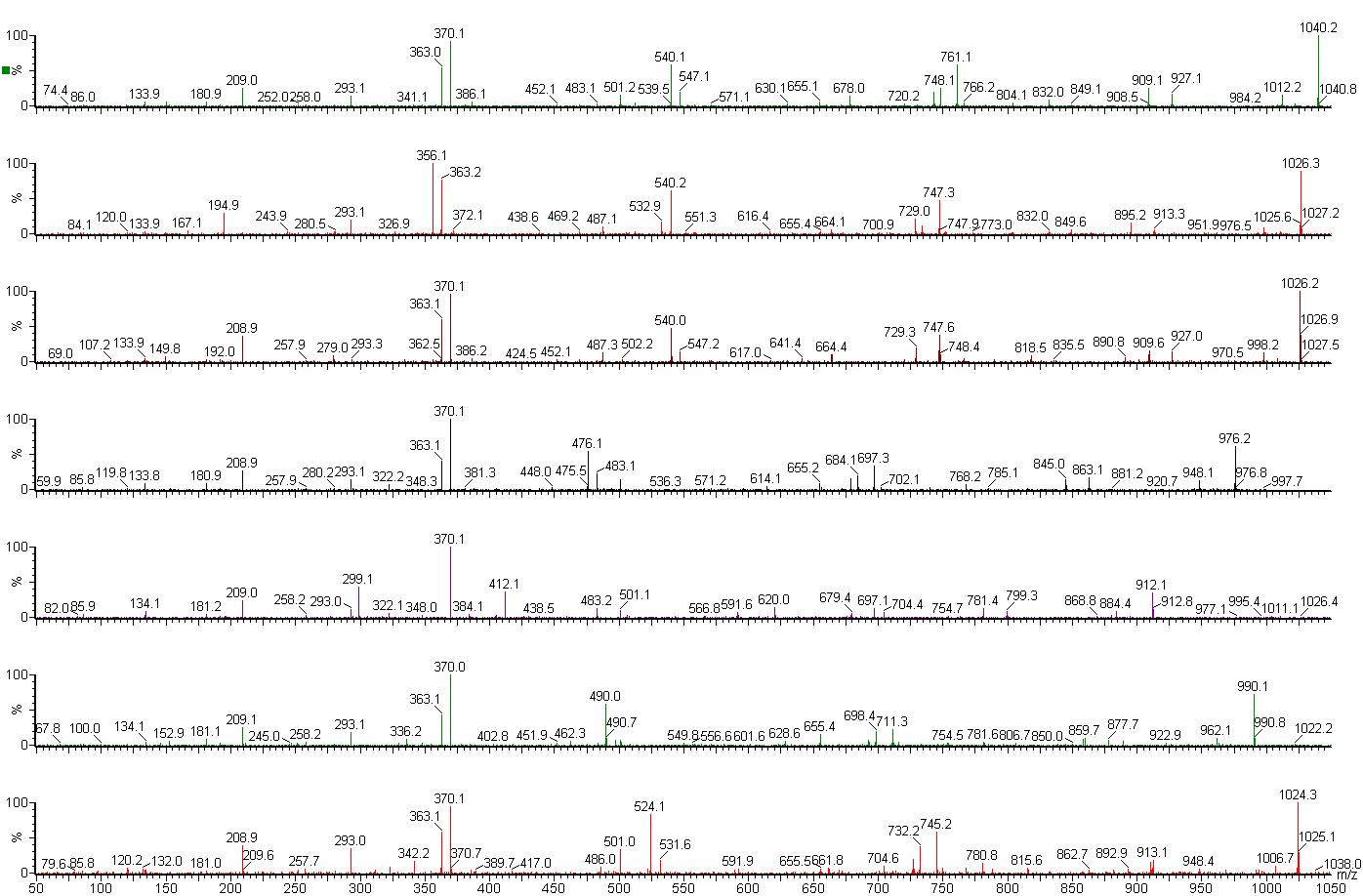
**

**Figure 2:** Results of mass spectrometric fragmentation experiments with NIVA CYA 116. Note that all fragmentation experiments were run with the [M+H -SO_4_ -H_2_O]^+^ ion, which is the most abundant ion under the experimental conditions used in this study. The identity of selected diagnostic fragments is given.


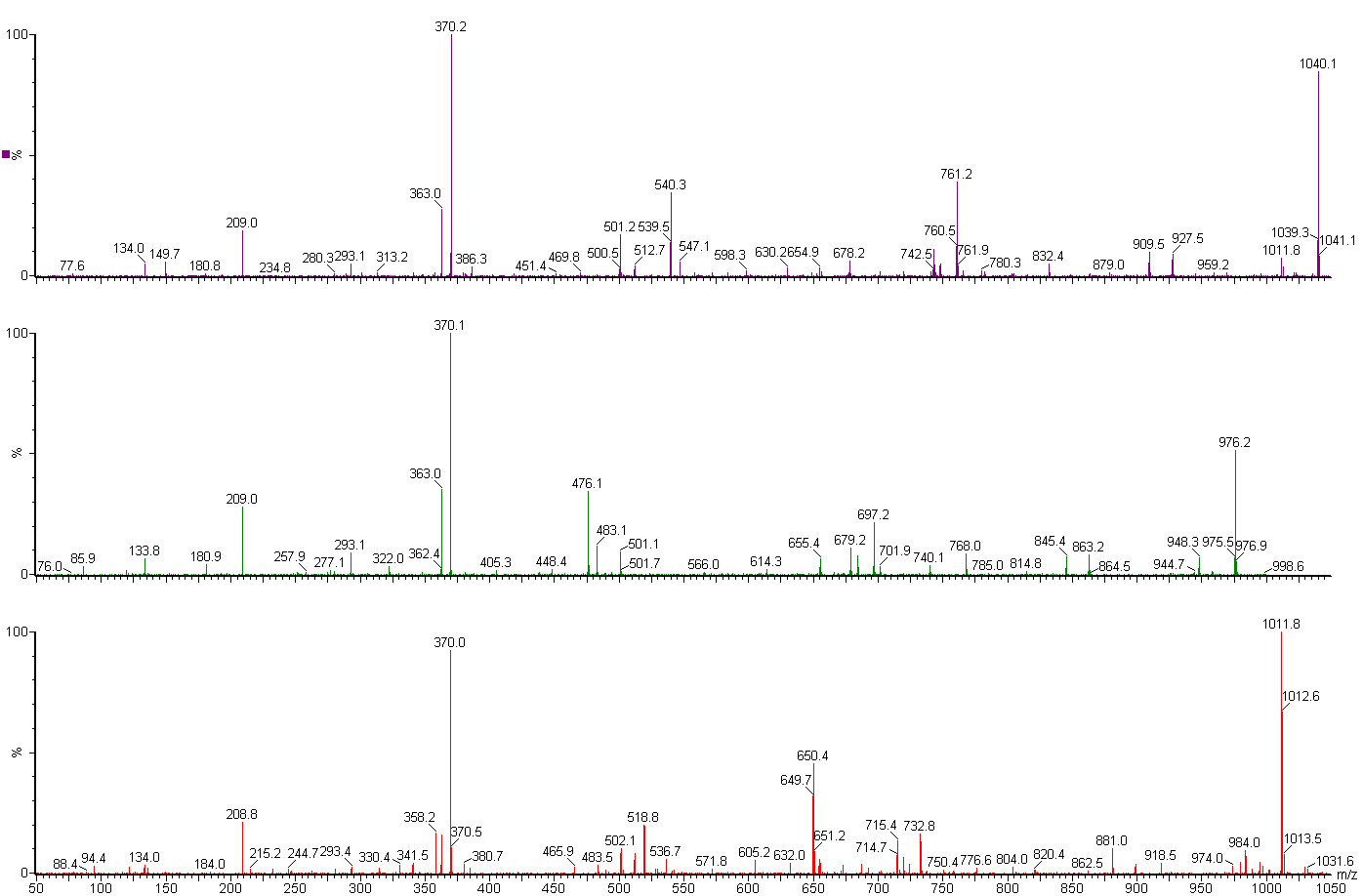


**Figure 3**: Results of mass spectrometric fragmentation experiments with NIES 205. Note that all fragmentation experiments were run with the [M+H -SO_4_ -H_2_O]^+^ ion, which is the most abundant ion under the experimental conditions used in this study. The identity of selected diagnostic fragments is given.


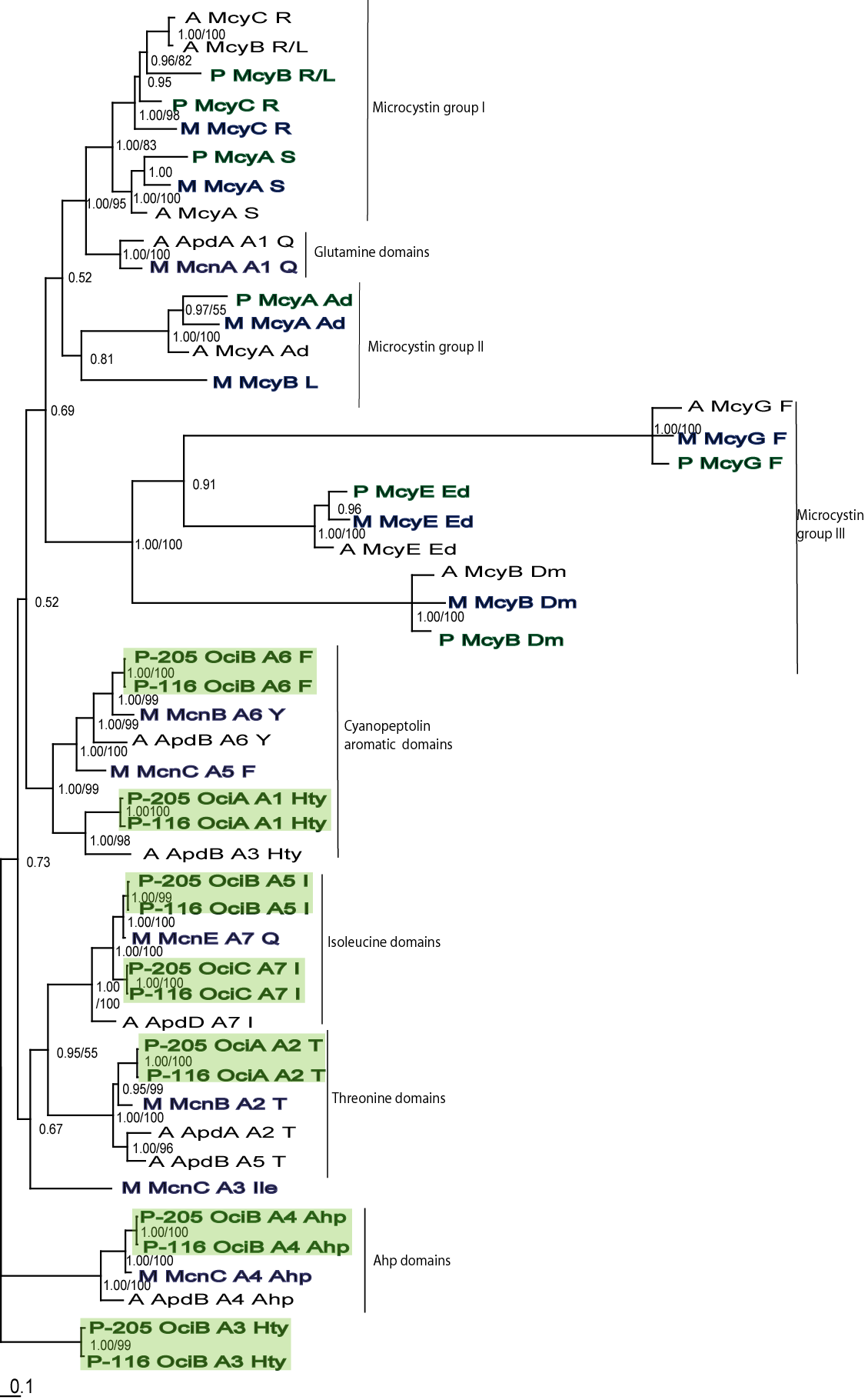


**Figure 4**: A-domain tree constructed using bayesian inference. Domains group by function (i.e. amino acid activated). Support values for the nodes are bayesian posterior probability and 1000 bootstrap replicates deduced from the NJ tree, above 50. The bayesian tree and the NJ tree showed nearly identical structures (except for a minor topology difference within the *Microcystis* group III). Genus origin is denoted with first letter abbreviations (P=*Planktothrix*, M=*Microcystis*, A=*Anabaena* and N=*Nostoc*) the putative activated amino acid shown in one letter abbreviations (Ad =D-alanine, Hty=homotyrosine, Ed=D- glutamate and Dm=methylaspartic acid). The WAG substitution model was used in the Bayesian analyses.


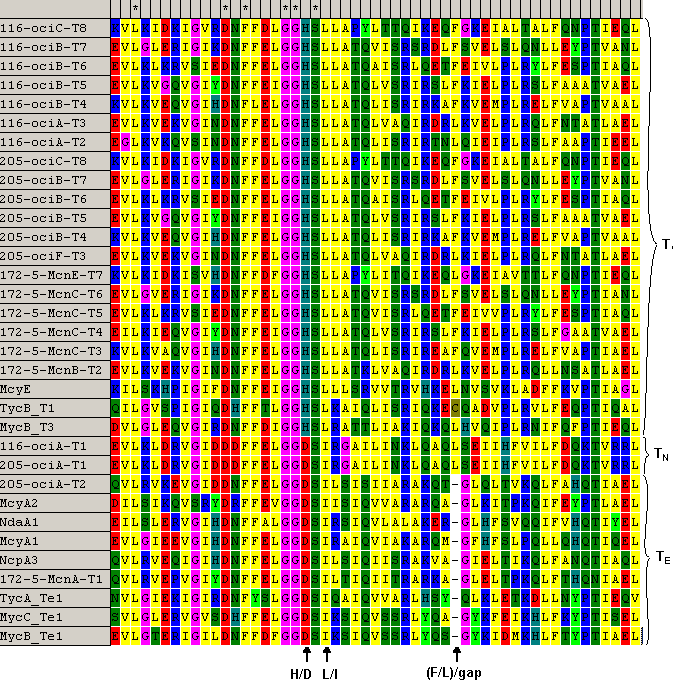


**Figure 5**: Protein alignment of partial T-domains from 116-Oci and 205-Oci from *Planktothrix*, Mcn and Mcy domains form Microcystis, Ncp domains from *Nostoc*, Nda from *Nodularia*, and Myc and Tyc domains from *Bacillus*. Invariant amino acids are shown with stars on top of the alignment. The transitions indicated in the alignment are indicative of T domains situated upstream of E-domains (T_E_). T_C_ domain is situated upsteam of C domains and T_N_ is N-terminal T-domains

**Table 1**: Accession numbers for *Planktothrix* NIVA CYA 116 and NIES 205

| Planktothrix | NIVA CYA 116 | NIES 205 |
| --- | --- | --- |
| 16S rDNA | AB045911 | AB045955 |
| ntcA | EU109508 | EU109505 |
| *cpc*BA spacer | EU109507 | EU109506 |
| *oci* gene cluster | DQ837301 | EU109504 |
